# A turn-on fluorescent probe with a dansyl fluorophore for hydrogen sulfide sensing†

**DOI:** 10.1039/c9ra04790e

**Published:** 2019-09-03

**Authors:** Yehan Yan, Lijuan Chen, Renyong Liu, Yu Zheng, Suhua Wang

**Affiliations:** West Anhui University Luan Anhui 237012 China; School of Environmental Science and Engineering, North China Electric Power University Beijing 102206 China wangsuhua@ncepu.edu.cn

## Abstract

Hydrogen sulfide (H_2_S) is a biologically relevant molecule that has been newly identified as a gasotransmitter and is also a toxic gaseous pollutant. In this study, we report on a metal complex fluorescent probe to achieve the sensitive detection of H_2_S in a fluorescent “turn-on” mode. The probe bears a dansyl fluorophore with multidentate ligands for coordination with copper ions. The fluorescent “turn-on” mode is facilitated by the strong bonding between H_2_S and the Cu(ii) ions to form insoluble copper sulfide, which leads to the release of a strongly fluorescent product. The H_2_S limit of detection (LOD) for the proposed probe is estimated to be 11 nM in the aqueous solution, and the utilization of the probe is demonstrated for detecting H_2_S in actual lake and mineral water samples with good reproducibility. Furthermore, we designed detector vials and presented their successful application for the visual detection of gaseous H_2_S.

## Abstract 2

H_2_S turn on the fluorescence of DNS–Cu complex probe.

## Graphical abstract

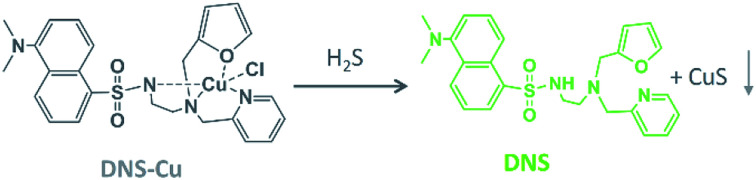

## 1. Introduction

Hydrogen sulfide (H_2_S) is a highly toxic air pollutant that is widely generated from industrial production processes, involving sulfuric acid, sulfur, dyes, and cosmetics, as well as the microbial degradation processes by which anaerobic bacteria reduce inorganic sulfates and organic sulfides.^1^ Prolonged exposure to H_2_S may lead to respiratory paralysis, olfactory fatigue, vagueness of consciousness, and even permanent cerebral injury.^2,3^ In addition, H_2_S is a newly identified endogenous gaseous transmitter molecule like NO and CO, which has been associated with the regulation of cardiovascular, vasodilation, central nervous, respiratory, and immune systems.^4–10^ The level of H_2_S in humans has been documented to be between 10 and 100 μM, and an abnormal concentration of H_2_S has been related with Down syndrome, diabetes, Alzheimer's disease, and arterial and pulmonary hypertension.^11–13^ Therefore, analytical approaches capable of conducting selective and sensitive H_2_S detection in complicated environmental and biological systems are essential.

Current techniques employed for H_2_S analysis include chemical titration,^14,15^ colorimetry,^16^ electrochemical assay,^17^ gas chromatography,^18^ and inductively coupled plasma atomic emission spectroscopy (ICP-AES).^19^ Compared to these methods, fluorescence analysis has proven to be a promising option because of its high sensitivity, high temporal and spatial detection resolutions, and the ability for conducting *in situ* monitoring of reactive and transient target analytes.^20–27^ Generally, the fluorescence probes for H_2_S mainly based on reduction reactions,^28–32^ nucleophilic addition reactions,^33–35^ and thiolysis reactions.^36–38^ In addition, metal displacement approach utilizes the strong affinity of metal ions with sulfides ions to rapidly attain reaction equilibrium, and achieve the real-time detection of H_2_S.^39,40^

Based on metal displacement approach of copper metal complexes, some colorimetric or fluorescent sensors for H_2_S have been emerging in recent years, owning to the low solubility product of CuS (*K*_sp_ = 6.3 × 10^−36^). For example, Li *et al.* synthesized a Cu-complex probe (BODIPY–DPA–Cu) by attaching di-(2-picolyl)amine (DPA) and BODIPY dye, in the presence of H_2_S, the fluorescence at 546 nm enhanced 19-fold in the PBS buffer (10 mM, pH 7.4). It showed a high sensitivity and selectivity for sulfide.^41^ Kim J. Y. and co-workers developed a Cu-cyclen-dansyl (Cu-CD) probe for the quantification of H_2_S in PBS buffer containing 10% DMSO with good selectivity among competitive anions.^42^ Kaushik and co-workers designed a probe for selective detection of H_2_S by copper complex embedded in vesicles, different from the MDA mechanism, in which both metal and indicator get displaced upon binding of H_2_S with metal center.^43^ Other relevant probes for H_2_S detection based copper metal complexes are listed in Table S1.†^44–51^

The present work capitalizes on this property by combining a dansyl fluorophore with a Cu(ii) metal complex to design a displacement-reaction-based probe for H_2_S detection in a fluorescent “turn-on” mode. The fluorescent metal complex probe includes a multidentate ligand that can coordinate with Cu^2+^ ions to produce a stable dansyl fluorophore Cu-complex (DNS–Cu). The DNS–Cu complex exhibits a very weak background fluorescence because the empty d orbital of paramagnetic Cu(ii) can accept the excited state electrons in the DNS fluorophore, and hence, block the process of ligand fluorescence generation. In addition, H_2_S strongly binds with the Cu^2+^ ions to form CuS and releases a free fluorescent DNS fluorophore, resulting in a greatly enhanced probe fluorescence. The H_2_S limit of detection (LOD) for the proposed probe (DNS–Cu) is determined to be 11 nM, and the probe demonstrates good H_2_S selectivity, reproducibility, and anti-interference performance. In addition, the probe was used to develop detector vials for visualizing gaseous H_2_S, which demonstrates the applicability of the proposed fluorescent probe for sensing H_2_S gas in the environment.

## 2. Experimental

### 2.1 Materials

All of reagents were received from commercial suppliers. Sodium sulfide (Na_2_S), sodium nitrate (NaNO_3_), sodium sulfate (Na_2_SO_4_), sodium thiocyanate (NaSCN), sodium thiosulfate (Na_2_S_2_O_3_), sodium fluoride (NaF), sodium chloride (NaCl), sodium nitrate (NaNO_3_), sodium nitrite (NaNO_2_), sodium bromide (NaBr), potassium iodide (KI), sodium sulfite (Na_2_SO_3_), sodium hypochlorite (NaClO), trisodium phosphate (Na_3_PO_4_), copper(ii) chloride dihydrate (CuCl_2_·2H_2_O), reduced glutathione (GSH), l-cysteine (l-Cys), *N*,*N*-dimethyl formamide, ethyl acetate (EA), petroleum ether (PE), dichloromethane, anhydrous potassium carbonate, 5-dimethylamino-1-naphthalenesulfonyl chloride, triethylamine, methanol and 2-chloroethylamine hydrochloride. Adenosine 5′-diphosphate disodium salt (ADP), adenosine 5′-triphosphate disodium salt (ATP), adenosine 5′-monophosphate (AMP), pyrophosphoric acid (PPi), the ultrapure water (18.2 MΩ cm) was used throughout the experiments.

### 2.2 Instrumentation

The fluorescence spectra were obtained on a PerkinElmer LS-55 luminescence spectrometer. Mass spectra were recorded on a Thermo Proteome X-LTQ MS. ^1^H-NMR spectrum was acquired on a Brucker Avance 400 MHz, using CDCl_3_ as solvent and tetramethylsilane (TMS) as internal standard. Fluorescence photos were taken under a UV lamp, with a Canon 350D digital camera.

### 2.3 Synthesis of the dansyl fluorophore and its Cu(ii) metal complex (DNS–Cu)

**Scheme 1:**
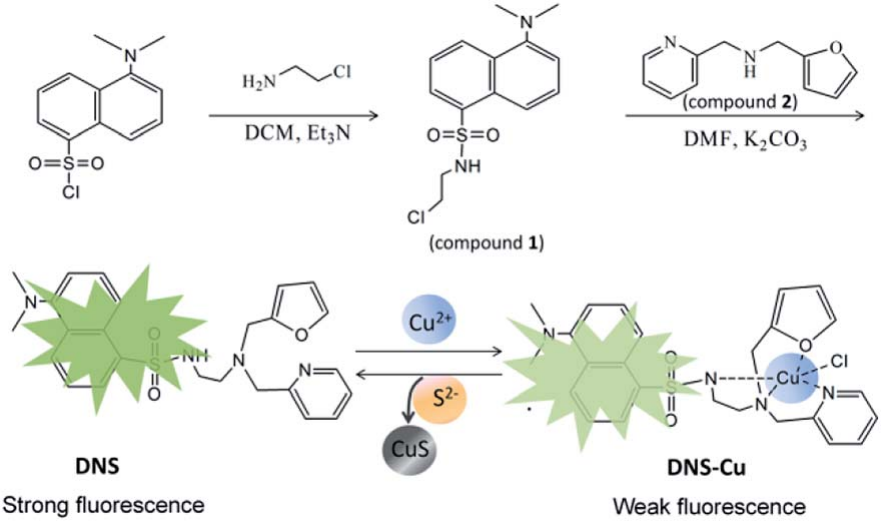
The scheme shows the synthetic procedure of DNS and its copper complex (DNS–Cu) probe. The DNS–Cu complex probe can combine with S^2−^ to generate a very stable CuS precipitation and release the DNS fluorophore.

The synthesis processes of DNS and the DNS–Cu complex were illustrated in Scheme 1. Firstly, we added 2-chloroethylamine hydrochloride (65 mg) to a dichloromethane (10 mL) solution of dansyl chloride (150 mg) and triethylamine (Et_3_N; 117 μL). After stirring for 1 h, a pale-yellow oil was obtained by removing the solvent, and then purified *via* column chromatography with a PE : EA ratio of 4 : 1. The resulting product is herein denoted as compound 1 (107 mg, 61%). For the synthesis of DNS, we added 62.5 mg of compound 1, 37.6 mg of compound 2, and 27.6 mg of anhydrous K_2_CO_3_ into 3 mL of a dimethylformamide (DMF) solution. After stirring the mixture for 24 h, 15 mL of ice water was poured into the mixture to generate a precipitate. The solid crude product was collected through filtration, and the desired pale yellow oil DNS product (40 mg, 43.1%) was obtained by column chromatography using PE : EA ratios from 4 : 1 to 1 : 1. The DNS–Cu probe complex was easily synthesized by mixing equal molar amounts of DNS and CuCl_2_·2H_2_O solutions in C_2_H_5_OH/H_2_O (1 : 1, v/v). The structures of the DNS and DNS–Cu complex were both characterized by mass spectrometry (MS). Electrospray ionization (ESI) MS (ESI-MS) of DNS (*m*/*z*): calculated for 464.19, found 465.19 (M + H^+^), as shown in Fig. S1 in the ESI† and its proton nuclear magnetic resonance (^1^H-NMR; Fig. S2,† 400 MHz, CDCl_3_). ^1^H-NMR (400 MHz, CDCl_3_) *δ* 8.56–8.51 (m, 1H), 8.43 (d, *J* = 8.5 Hz, 1H), 8.32 (d, *J* = 8.7 Hz, 1H), 7.53 (td, *J* = 7.7, 1.8 Hz, 1H), 7.45–7.39 (m, 1H), 7.36 (dd, *J* = 8.6, 7.6 Hz, 1H), 7.21 (dd, *J* = 1.8, 0.8 Hz, 1H), 7.13–7.03 (m, 3H), 6.69 (s, 1H), 6.15 (dt, *J* = 9.9, 4.9 Hz, 1H), 5.94 (d, *J* = 2.9 Hz, 1H), 3.56 (s, 2H), 3.20 (s, 2H), 2.82–2.92 (m, 2H), 2.75 (s, 6H), 2.53 ppm (t, *J* = 4 Hz, 2H).

### 2.4 Determination of the binding constant of the DNS–Cu complex

The stability constant *K* of the DNS–Cu complex was calculated from a Benesi–Hildebrand plot according to the following Benesi–Hildebrand equation.^52,53^
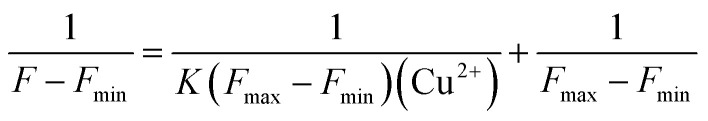
Here, *F*_max_ is the fluorescence intensity of the free DNS, *F* is the fluorescence intensity of DNS with Cu^2+^, *F*_min_ stands for the fluorescence intensity of DNS in the presence of excessive Cu^2+^

### 2.5 Measurement of quantum yields (QYs) of the DNS–Cu complex

The QY was measured by using fluorescein (*Φ*_s_ = 0.95 in 0.1 M NaOH) as reference and calculated using the following equation.
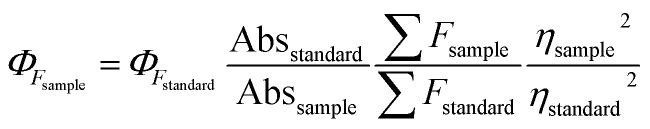
where *Φ*_F_ stands for the fluorescence quantum yields, Abs and ∑*F* denote the absorbance and the measured integrated fluorescence intensity at the excitation wavelength, and *η* is the refractive index of the solvent used. The refractive index of H_2_O and C_2_H_5_OH/H_2_O (1 : 1, v/v) mixture solvent was 1.33 and 1.35, respectively.

### 2.6 Detection sensitivity of the DNS–Cu complex probe for H_2_S

Firstly, 2.0 μL of the DNS–Cu probe solution (1.0 mM) was added to 2.0 mL of a C_2_H_5_OH/H_2_O (1 : 1, v/v) solution, yielding a 1.0 μM DNS–Cu concentration in the probe solution. The Na_2_S solution was freshly prepared used as the H_2_S source in the aqueous solution. The added concentrations of sulfur ion (S^2−^) in the probe solution were 0, 0.125, 0.250, 0.375, 0.500, 0.625, 0.750, 0.875, 1.000, 1.125, 1.250, and 1.375 μM. The fluorescence spectra were obtained between the range of 400 nm and 700 nm under an excitation wavelength of 338 nm. The primary fluorescence peak of the DNS–Cu probe obtained at 534 nm was employed for S^2−^ sensitivity testing. The fluorescence ratio *F*/*F*_0_ of the DNS–Cu probe, where *F*_0_ is the value of *F* obtained with no added S^2−^ ions, was plotted *versus* the S^2−^ ions concentration for quantitative analysis. All data were performed three times under equivalent conditions, and the average values are calculated.

### 2.7 Detection selectivity of the DNS–Cu complex probe for H_2_S

The selective responses of the DNS–Cu probe for other related anions and small molecules containing thiol groups were carefully examined using the same testing procedure as was employed for S^2−^. The stock solutions of these species (1 mM of NO_3_^−^, NO_2_^−^, SO_4_^2−^, SO_3_^2−^, SCN^−^, S_2_O_3_^2−^, F^−^, Cl^−^, Br^−^, I^−^, PO_4_^3−^, and ClO^−^) were prepared in ultrapure water.

To further illustrate the practical application of the proposed probe, anti-interference experiments were conducted by adding NO_3_^−^, NO_2_^−^, SO_4_^2−^, or SO_3_^2−^ (50 equiv.), and SCN^−^, S_2_O_3_^2−^, F^−^, Cl^−^, Br^−^, I^−^, PO_4_^3−^, or ClO^−^ (10 equiv.) into the DNS–Cu probe (1.0 μM) solution. Then, 1 equiv. of S^2−^ ions (1.0 μM) was added into the mixture solution, followed by recording the fluorescence intensity at 534 nm.

The selectivity of the DNS–Cu probe was also examined for H_2_S and other different common gases. Gaseous H_2_S was obtained by slowly dropping phosphoric acid on sodium sulfide. Carbon monoxide (CO) gas was achieved from the reaction of formic acid with concentrated sulfuric acid. Carbon dioxide (CO_2_) gas was prepared from the titration reaction between dilute sulfuric acid and sodium bicarbonate. Sulfur dioxide (SO_2_) was got through a quantitative reaction of sodium hydrosulfide and concentrated sulfuric acid. Ammonia (NH_3_) gas was generated through the chemical reaction between NH_4_Cl and Ca(OH)_2_. Nitric oxide (NO) and nitrogen dioxide (NO_2_) gas were obtained from pure gas. Then, different of these gas samples were injected into the DNS–Cu probe solution using a syringe, recording the fluorescence spectra subsequently and taking the fluorescence photos by a digital camera.

## 3. Results and discussion

### 3.1 Characterization of DNS and DNS–Cu

The structure of the DNS confirmed by the analysis of MS and ^1^H-NMR (Fig. S1 and S2†). As illustrated in Scheme 1, the DNS fluorophore exhibits a bright fluorescence at 534 nm, after coordinating with Cu^2+^, DNS–Cu displays a very weak fluorescence (Fig. S3†). The MS analysis verified the formation of the DNS–Cu complex. ESI-MS of DNS–Cu (*m*/*z*): calculated 526.1100 found 526.0400 (Fig. S4†). The value of stability constant (*K*) for the DNS–Cu complex was determined to be 2.7 × 10^4^ based on Benesi–Hildebrand method, as seen in Fig. S5.†

### 3.2 Stability and sensitivity of the DNS–Cu probe for H_2_S

**Fig. 1:**
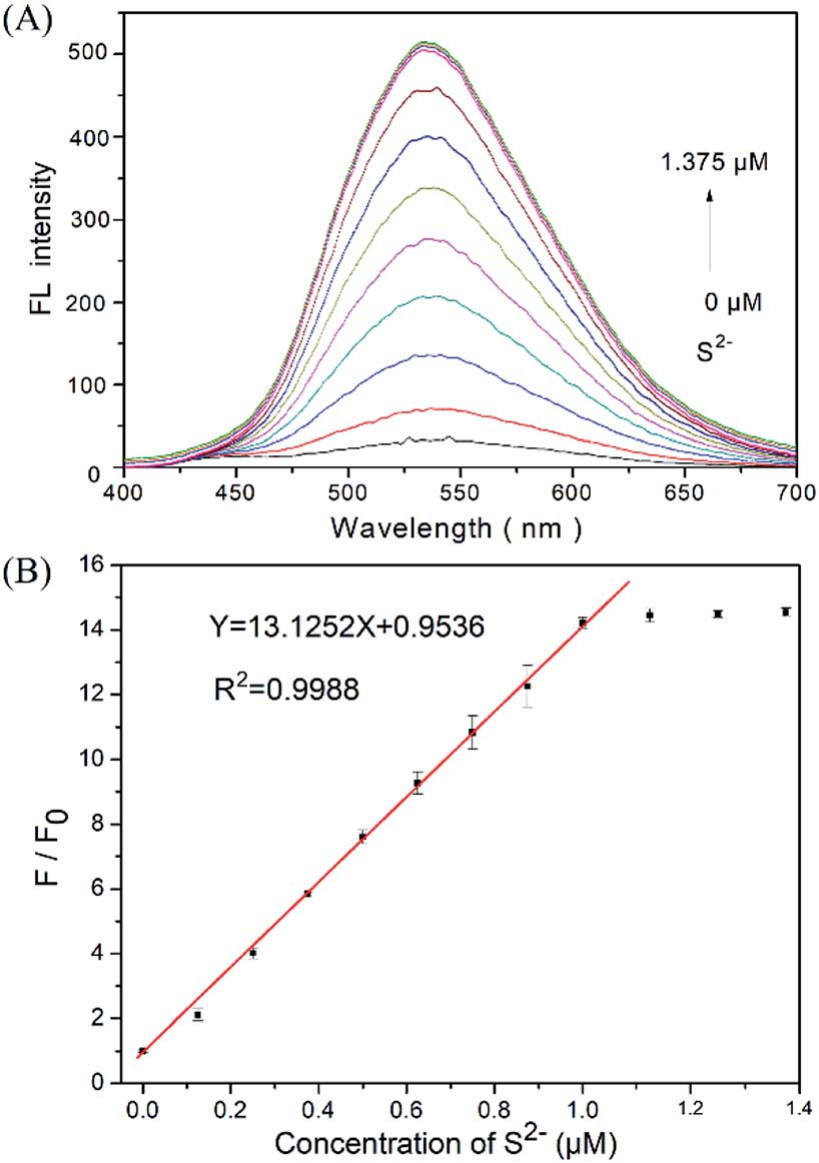
(A) Fluorescence of the DNS–Cu probe solution (1.0 μM) in the presence of different concentrations of S^2−^ ions. (B) Plot of the fluorescence ratio *F*/*F*_0_ of the probe with respect to the S^2−^ concentration, where *F*_0_ and *F* represent the fluorescence intensity of the DNS–Cu probe at 534 nm without and with the addition of S^2−^ ions, respectively.

We firstly investigated the stability of the DNS–Cu complex probe before the sensitivity experiment. As noted from Fig. S6,† the fluorescence intensity of the probe at 534 nm exhibits no distinct changes after six consecutive irradiations for 60 min, indicating that the DNS–Cu complex probe is stable against photobleaching. When the probe solution (1.0 μM) is exposed to S^2−^ ions, as seen in Fig. 1A, the fluorescence intensity of the probe greatly increased as the S^2−^ ions concentration increased from 0 to 1.375 μM. This can be attributed to the strong binding of S^2−^ with the Cu(ii) metal center of the DNS–Cu complex to form stable CuS precipitation, subsequently releases the DNS fluorophore. The MS and ^1^H-NMR analysis proved the release of the DNS in Fig. S7,† and the quantum yields were increased from 1.8% to 25.5% after the DNS–Cu probe reaction with S^2−^(Fig. S8†). In addition, the fluorescence intensity exhibits a dose–response with increasing S^2−^ concentration up to an S^2−^ dose of 1.0 μM, after which further increases in the S^2−^ concentration produce no further increase in the fluorescence intensity. This indicates that the stoichiometric reaction between the DNS–Cu probe and S^2−^ was 1 : 1. The plot of *F*/*F*_0_*versus* the S^2−^ concentration in Fig. 1B exhibits a good linear relationship with a coefficient of determination *R*^2^ = 0.9988 in the S^2−^concentration range of 0 to 1.0 μM. The LOD was estimated to be 11 nM based on the definition of LOD = 3 × S.D./*k*, where *k* is the slope of the curve in Fig. 1B, and 3 × S.D. stands for 3 times standard deviation of the blank signal.

### 3.3 Selectivity of the DNS–Cu probe for H_2_S

**Fig. 2:**
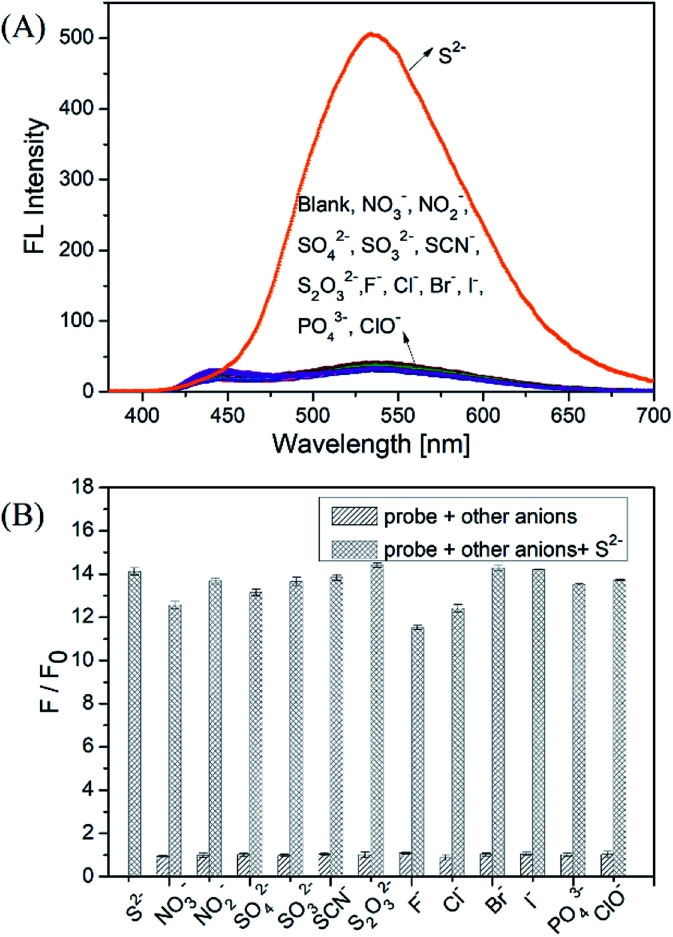
(A) Fluorescence spectra of the DNS–Cu probe solution (1.0 μM) for S^2−^ ions (1.0 μM) and other anion species (1.0 μM). (B) *F*/*F*_0_ values at 534 nm of the probe to various anions. The sparse bars represent the fluorescence responses of the probe in the presence of other anions (50 equiv. of NO_3_^−^, NO_2_^−^, SO_4_^2−^, SO_3_^2−^ and 10 equiv. of SCN^−^, S_2_O_3_^2−^, F^−^, Cl^−^, Br^−^, I^−^, PO_4_^3−^ and ClO^−^), the dense bars represent the subsequent addition of 1 equiv. of S^2−^ (1.0 μM) into the mixture solution.

As shown in Fig. 2A, the fluorescence intensity of the DNS–Cu probe solution increased sharply after the addition of S^2−^ (1.0 μM), whereas no apparent changes in the fluorescence intensity were observed after adding the NO_3_^−^, NO_2_^−^, SO_4_^2−^, SO_3_^2−^, SCN^−^, S_2_O_3_^2−^, F^−^, Cl^−^, Br^−^, I^−^, PO_4_^3−^, and ClO^−^ anionic species (1.0 μM), indicating a high selectivity for S^2−^ ions. For further practical application, the anti-interference experiments of the probe were conducted by adding NO_3_^−^, NO_2_^−^, SO_4_^2−^, SO_3_^2−^ (50 equiv.), and SCN^−^, S_2_O_3_^2−^, F^−^, Cl^−^, Br^−^, I^−^, PO_4_^3−^ ClO^−^ (10 equiv.) into the probe solution, respectively. Then 1 equiv. of S^2−^ ions (1.0 μM) were added subsequently into the mixture solution, followed by recording the fluorescence intensity at 534 nm. The results of the anti-interference experiments shown in Fig. 2B, indicating that the probe exhibits a good anti-interference against other anionic species.

In order to verify the practicability of probe in complex environments, some biological phosphates (PPi, ATP, ADP, AMP) and endogenous biomolecule (GSH, l-Cys) were investigated with DNS–Cu probe. The results show that the PPi, ATP, ADP and AMP could not turn on the fluorescence of the probe system (Fig. S9†). Even in the presence of 10 equiv. of PPi, ATP, ADP and AMP (10 μM), H_2_S (1.0 μM) also can enhance the fluorescence of the DNS–Cu probe in the same manner as in the absence of these analytes. These results suggest that biological phosphates dose not interference the detection system. However, it is noted that GSH and cysteine could enhance the fluorescence of the DNS–Cu probe, which may be attributed to the affinity of Cu^2+^ with –SH groups. Fortunately, their interference with these thiol-containing molecules can be readily eliminated through treatment with dimethyl sulfoxide (DMSO), which oxidizes the thiol groups to form disulfides that have less affinity with Cu(ii), which minimizes their interference effect (Fig. S10†).

### 3.4 Potential reusability of the DNS–Cu probe

**Fig. 3:**
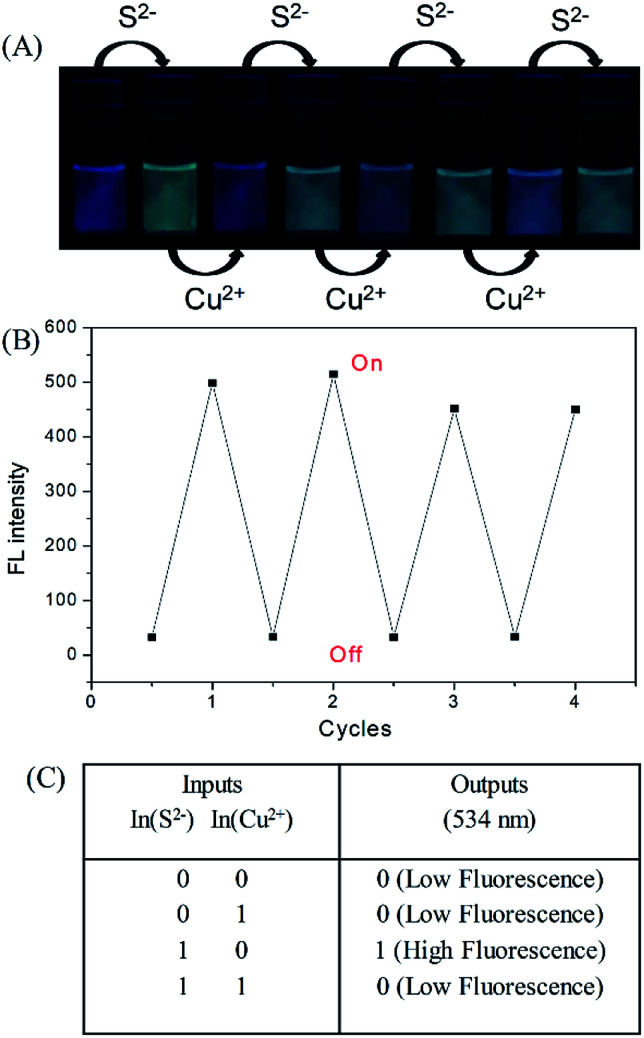
Fluorescence images (A) and fluorescence intensity (B) of the DNS–Cu probe solution with the alternate addition of S^2−^ and Cu^2+^. The photographs were recorded under an ultraviolet (UV) lamp. (C) Logic table of an INHIBIT logic gate.

The fluorescence of the DNS–Cu probe can be activated by the addition of S^2−^, quenched by the addition of 1 equiv. of Cu(ii), and then recovered again by adding another 1 equiv. of S^2−^. As shown in Fig. 3, such fluorescence on–off cycles with S^2−^ and Cu(ii) could be repeated for 4 times with little degradation in the fluorescence intensity (Fig. 3B), and their corresponding fluorescent images recorded in Fig. 3A. The good reusability of the fluorescent probe for the alternate detection of S^2−^ and Cu^2+^ can be developed as a logic gate by operating the two inputs In(S^2−^) and In(Cu^2+^), as indicated in Fig. 3C. Optical logic gates, such as the YES,^54^ NOT,^55^ AND,^56^ OR,^57^ NOR,^58^ XOR,^59^ and INHIBIT gates^60^ have been investigated widely in recent years. Here, the two inputs can be set as 0 and 1 to represent the absence and presence of fluorescence, respectively, where exposure to S^2−^ results in a fluorescence on state, and the output readout is 1, while the fluorescence would be turned off by exposure to Cu^2+^ ions, leading to an output of 0. When there are no inputs of H_2_S and Cu^2+^ to the initial logic solution of the probe DNS–Cu complex, it shows no fluorescence, then the output signal is 0. While both of H_2_S and Cu^2+^ exist at the same time, it still no fluorescence, the output signal is 0 as well. Briefly, no fluorescence results from both inputs set as 0 or 1 simultaneously, the output signals were both 0, which match with the INHIBIT logic function.

### 3.5 Application of the DNS–Cu probe to actual water samples

Spike and recovery experiments were conducted using actual lake and mineral water samples. The lake water samples were collected from Shushan lake and filtered through a 0.45 μm microporous filter to remove insoluble particles. The mineral water samples were bought from a local supermarket and used directly without any pretreatment. The original concentrations of S^2−^ in lake water and mineral water samples were firstly confirmed to be 4.2 nM and 5.3 nM by ICPMS (iCAP RQ). These values were much lower than the maximum allowable level of S^2−^ (15 μM) set by World Health Organization (WHO) for drinking water. Thus, they do not pose any health concerns. Then the spiked recovery tests were conducted with three different S^2−^ concentrations (0, 500, and 1000 nM) in a mixed solution of water/ethanol (v/v = 1 : 1). The average concentration and standard deviations of S^2−^ in the spiked lake and mineral water samples are presented in Table 1. The concentrations of sulfide estimated in the non-spiked lake and mineral water samples were less than the LOD of the method. The two values are lower than the detection limit, suggesting that the sensitivity of the method has its own limitation and cannot be directly used to real water samples with trace contents, but can be used in the further recovery calculation. The recovery results in the lake water samples are slightly greater than 100%, which may be attributed to the microbial degradation process. Generally, the recovery rates ranged from 98.2% to 102.3% for the lake and mineral water samples, which are statistically near 100%, and therefore validates the ability of the proposed probe for S^2−^ ions sensing in complex samples.

**Table 1:** Recovery tests of S^2−^ ions spiked in lake water and mineral water

Add S^2−^ concentration (nM)	Lake water		Mineral water	
	Found (nM)	Recovery (%)	Found (nM)	Recovery (%)
0	6.2		7.8	
500	508	101.6 ± 3.09	491	98.2 ± 1.03
1000	1023	102.3 ± 1.48	997	99.7 ± 2.14

### 3.6 Visualization of H_2_S gas using detector vials

**Fig. 4:**
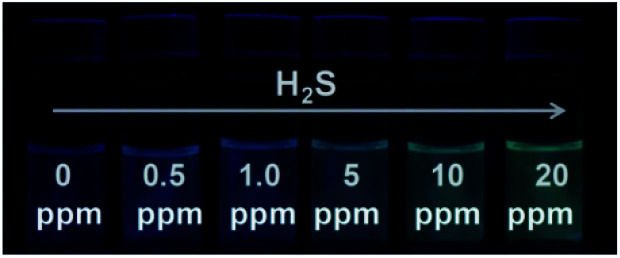
Visual detection of different concentrations of H_2_S gas under a UV light illumination in the dark using detector vials.

Since S^2−^ and H_2_S can quickly reach equilibrium in aqueous solution, this method can be applied to detect gaseous H_2_S in aqueous solution. For this measurement, we fabricated detector vials to achieve on-site and rapid detection of gaseous H_2_S. The vials itself has no fluorescence with a rubber stopper, different levels of H_2_S gas (0, 0.5, 1.0, 5.0, 10, and 20 ppm) were syringed into the vials. The images as presented in Fig. 4 indicate that the fluorescence color intensity in the vials increased with increasing H_2_S concentration. The limit of detection of this method was determined as 0.5 ppm, based on the minimum amount of H_2_S to produce a slightly different fluorescent color, which could be visible by five persons. The fluorescence intensity of the detector vial achieved a maximum level at an H_2_S concentration of 20 ppm.

**Fig. 5:**
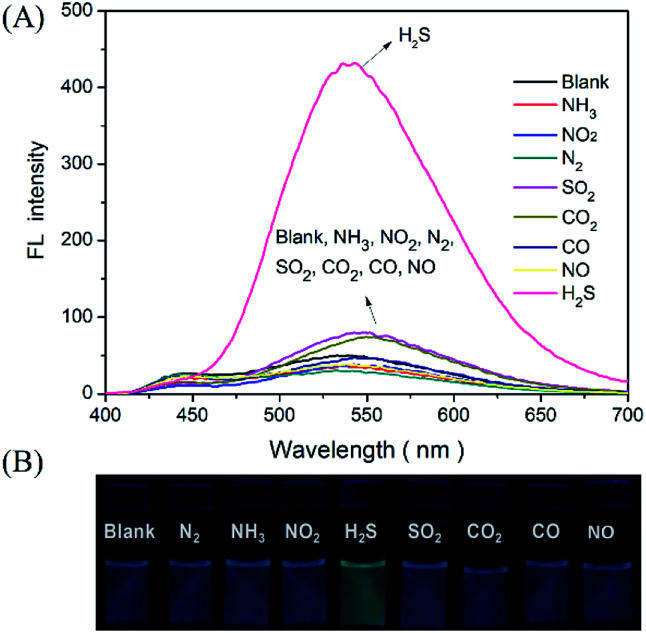
(A) Fluorescence spectra of the DNS–Cu probe solution for gaseous H_2_S. (B) The fluorescence images of the detector vials taken under a 365 nm UV lamp. The concentration of H_2_S gas was 20 ppm, N_2_, NH_3_, NO_2_, SO_2_, CO_2_, CO and NO was 100 ppm.

In addition, the selectivity of the proposed probe for H_2_S gas was investigated by evaluating the incidence of fluorescence when filling the detector vials with other common gases, such as N_2_, NH_3_, NO_2_, SO_2_, CO_2_, CO, and NO (100 ppm). As presented in Fig. 5. The results indicated that the injection of these other gaseous compounds has no substantial effect on the fluorescence intensity of the probe, and that only H_2_S gas activated the probe fluorescence. These results demonstrated a sensitivity and selectivity of this method for H_2_S determination in the gas state.

## 4. Conclusions

We have fabricated a dansyl-based copper complex for use as a “turn-on” fluorescence probe in H_2_S sensing. The subsequent addition of H_2_S effectively snatches Cu(ii) from the DNS–Cu complex, and thus releases a free dansyl moiety, leading to the fluorescence enhancement of the probe. The LOD for this method was determined to be 11 nM in aqueous solutions. In addition, the probe provided good H_2_S detection results with actual water samples. Moreover, the good reusability of the fluorescent probe for cyclical detection of H_2_S and Cu(ii), which can be used to develop “INHIBIT” logic circuit.

## Conflicts of interest

There are no conflicts to declare.

## Supplementary Material

RA-009-C9RA04790E-s001
