# Supplementary material for: A turn-on fluorescent probe with a dansyl fluorophore for hydrogen sulfide sensing
Source: RSC Adv. 2019 Sep 3;9(47):27652–8. doi: 10.1039/c9ra04790e (PMC9070855; doi:10.1039/c9ra04790e)
Supplement: RA-009-C9RA04790E-s001 [file RA-009-C9RA04790E-s001.pdf]

Electronic Supplementary Material (ESI) for RSC Advances.
This journal is © The Royal Society of Chemistry 2019

Electronic Supplementary Information

A turn-on fluorescent probe with a dansyl fluorophore for hydrogen sulfide sensing

Yehan Yan,[a] Lijuan Chen,[a] Renyong Liu,[a] Yu Zheng,[a] and Suhua Wang,*[,b]

[a]West Anhui University, Lu An, 237012, China.

[b]School of Environment Science and Engineering, North China Electric Power University, Beijing 102206, China.

Corresponding author:

E-mail address: wangsuhua@ncepu.edu.cn

Tel: 86-551-65591812

Table S1. The comparison of probe based copper metal complexes for H_2S. In terms of their solvent, excitation wavelength (Ex) and emission wavelength (Em) or the maximum absorption wavelength, limit of detection (LOD), cell imaging, H_2S gas test.

**NO.**	**Probe**	**Solvent**	**Ex/Em or A_{max} (nm)**	**LOD**	**Cell imaging**	**H_2S gas test**
1	Ves-1.Cu-EY	HEPES or PBS	515/540 nm or 564 nm	0.59 µM HEPES/ 4.06 µM PBS	Yes	No
2	naphthalimide–rhodamine B-Cu derivative colorimetric and fluorescent	$CH_3CN–H_2O$ (v/v = 7:3)	325/ 528 and 610 nm or 564 nm	0.23 µM	Yes	No
3	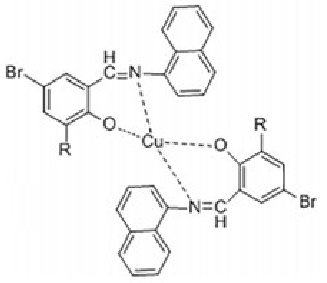 colorimetric and fluorescent	DMSO–H_2O (v/v = 9:1)	337/430 nm or shift 390 nm to 530 nm	0.13 µM	No	No
4	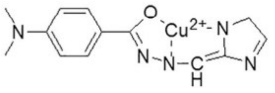 colorimetric and fluorescent	CH_3CN/H_2O (v/v = 3:2)	350/470 nm or shift 520 to 569 nm	0.68 µM	No	No
5	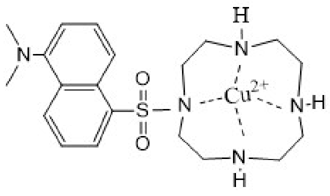	PBS (10% DMSO)	--/491 nm	4.07 µM	Yes	No

6	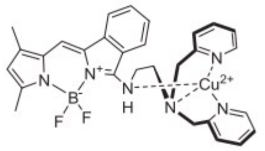	PBS pH 7.4	480 /546 nm	250 nM	Yes	No
7	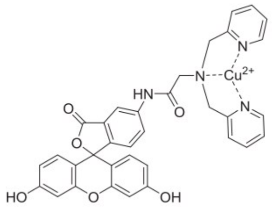	H_2O	470/517 nm	420 nM	No	No
8	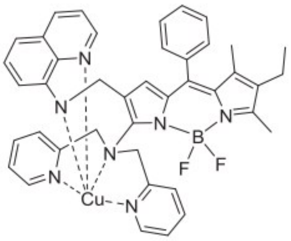 colorimetric	HEPES (10% DMSO)	shift 520 nm to 569 nm	167 nM	No	No
9	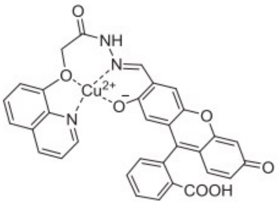	PBS/CH_3CN (1:1, v/v, pH 7.2)	495/534 nm	---	Yes	No
10	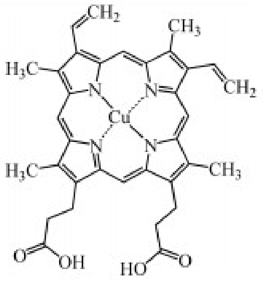	H_2O	386/623 and 682 nm	1 μM	NO	NO
11	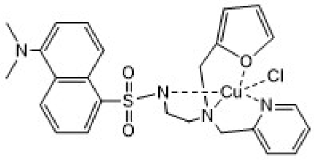 This work	C_2H_5OH/H_2O (v/v = 1:1)	338/534 nm	11 nM	No	Yes

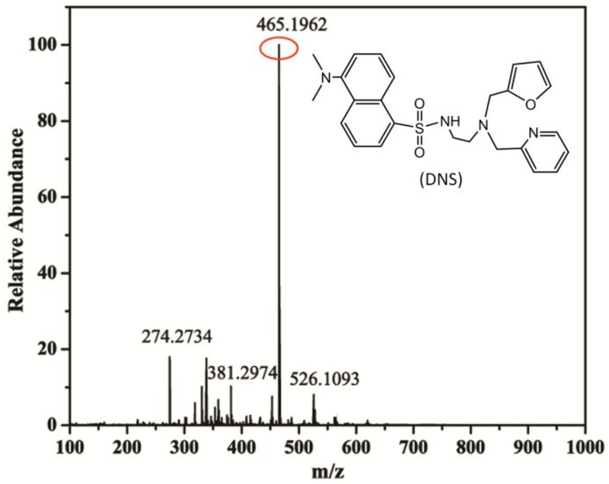

Fig. S1. ESI-Mass spectrum (positive mode) of DNS.

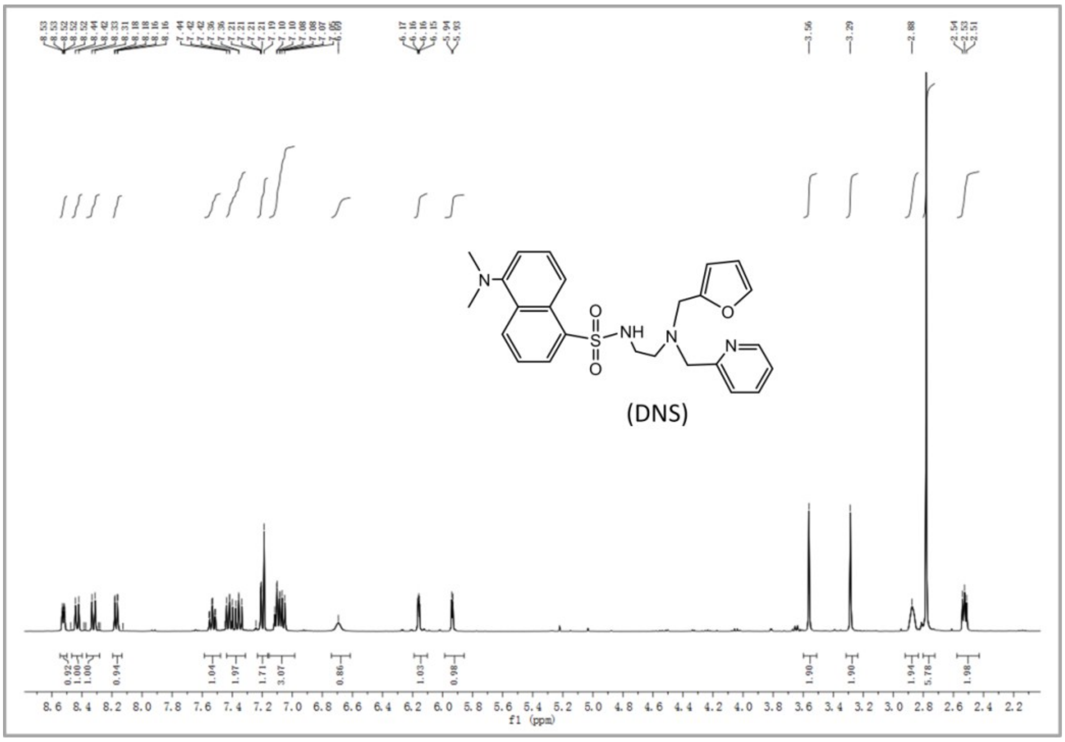

Fig. S2. ^{1}H-NMR spectrum of the DNS. ^{1}H-NMR (400 MHz, $CDCl_3$) δ 8.56-8.51 (m, 1H), 8.43 (d, J = 8.5 Hz, 1H), 8.32 (d, J = 8.7 Hz, 1H), 7.53 (td, J = 7.7, 1.8 Hz, 1H), 7.45-7.39 (m, 1H), 7.36 (dd, J = 8.6, 7.6 Hz, 1H), 7.21 (dd, J = 1.8, 0.8 Hz, 1H), 7.13-7.03 (m, 3H), 6.69 (s, 1H), 6.15 (dt, J = 9.9, 4.9 Hz, 1H), 5.94 (d, J = 2.9 Hz, 1H), 3.56 (s, 2H), 3.20 (s, 2H), 2.82-2.92 (m, 2H), 2.75 (s, 6H), 2.53 ppm (t, J = 4 Hz, 2H).

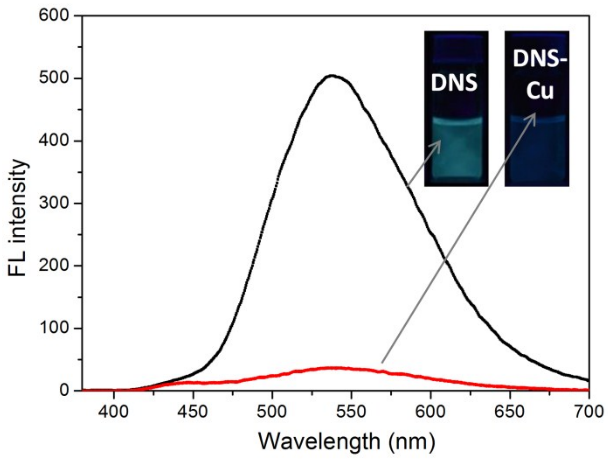

Fig. S3. The fluorescence spectra of DNS and the probe complex DNS-Cu. The insets are their corresponding photographs.

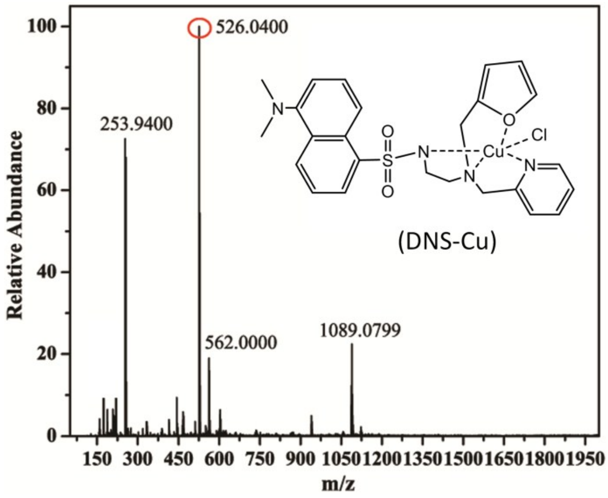

Fig. S4. The mass spectrum (positive mode) of DNS-Cu complex probe.

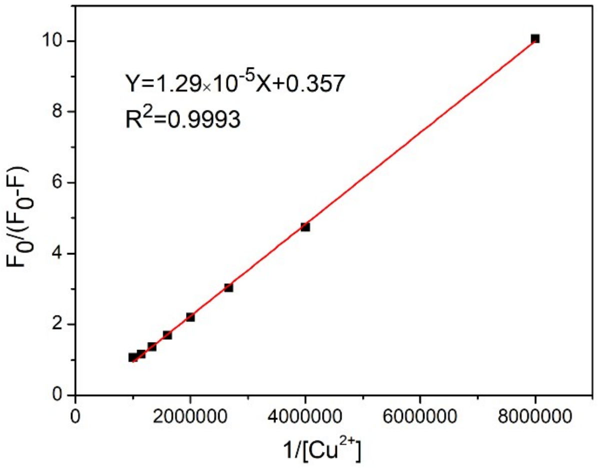

Fig. S5. Benesi-Hildebrand plot for determining the binding constant K of DNS-Cu complex.

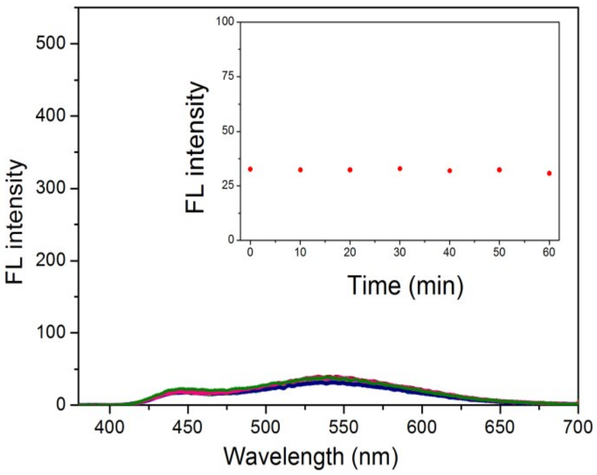

Fig. S6. The fluorescence spectra of probe recorded each 10 min for 1 h under ultraviolet irradiation at 338 nm. The fluorescence intensity has no significant change, implying probe exhibit good stability against photo-bleaching.

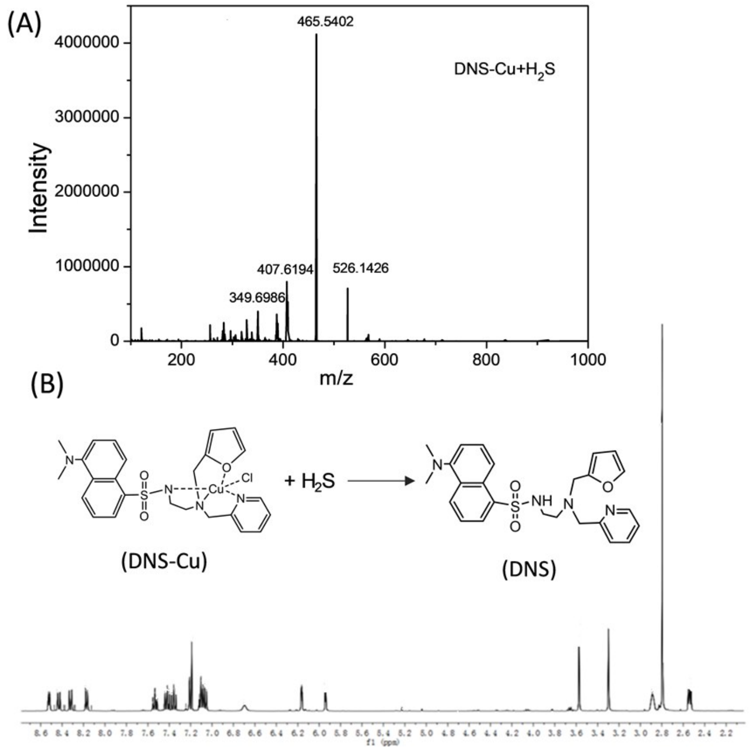

Fig. S7. (A) The mass spectrum (positive mode) and (B) ^{1}H-NMR spectrum (400 MHz, $CDCl_3$) of the DNS-Cu complex after reaction with H_2S. Clearly, the characteristic ^{1}H-NMR spectrum of DNS was restored.

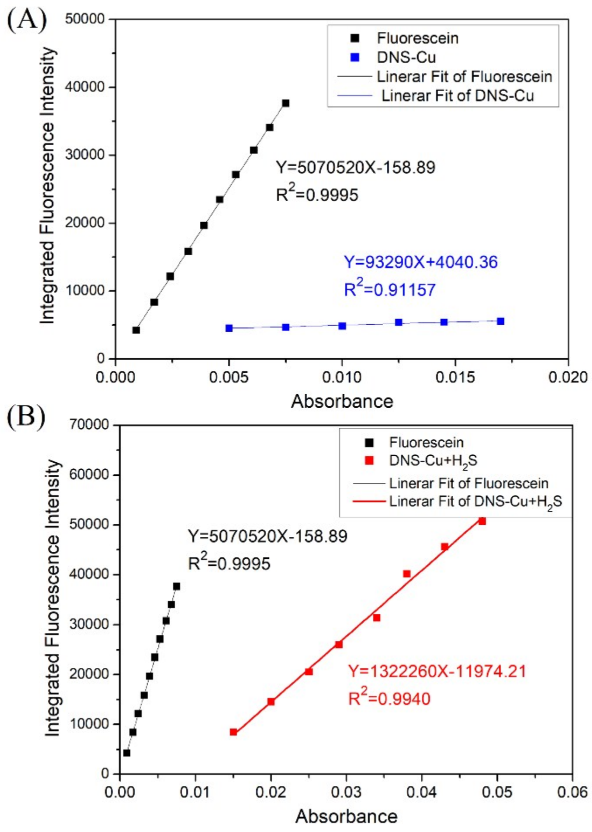

Fig. S8 Determination of the fluorescence quantum yield of (A) the DNS-Cu (QY, 1.8%) (B) after reaction with H_2S (QY, 25.5%), the fluorescein (Φs=0.95 in 0.1 M NaOH) as reference standard.

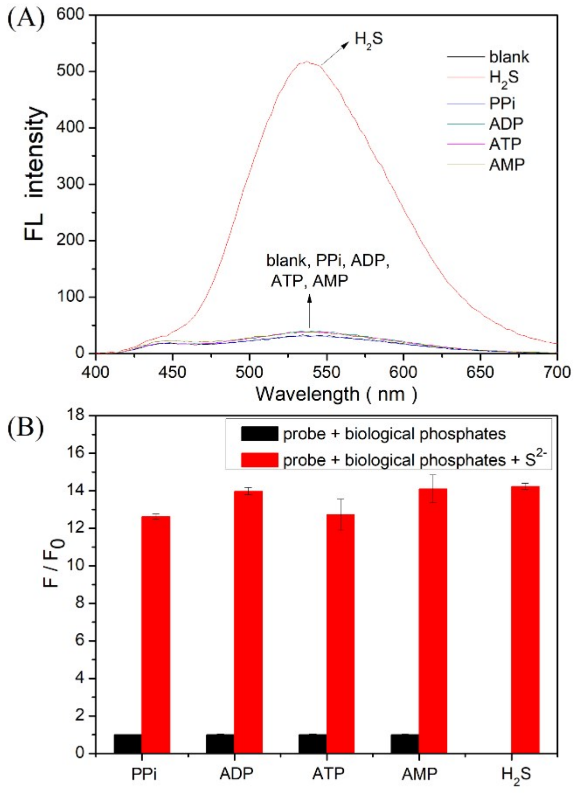

Fig. S9. (A) Fluorescence spectra of the DNS–Cu probe in presence of S^{2-} ions (1.0 μM) and other biological phosphates PPi, ATP, ADP and AMP (1.0 μM). (B) The effect of the biological phosphates on the fluorescence intensity of DNS–Cu complex. The black bars represent the probe in the presence of PPi, ATP, ADP and AMP, the red bars represent the subsequent addition of S^{2-}into the mixture solution. The concentration of S^{2-} was 1.0 μM, and the concentration of other species was 10 μM.

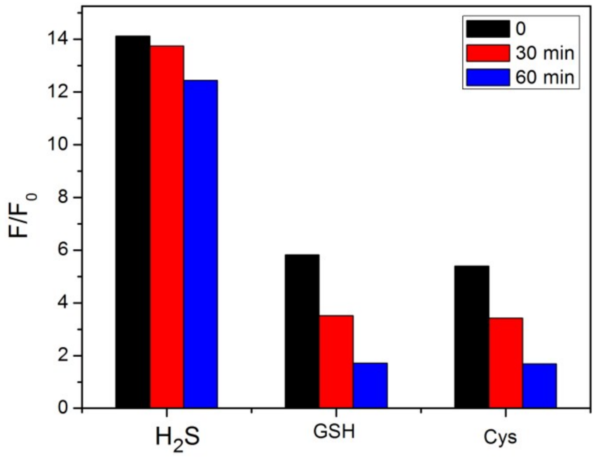

Fig. S10. Fluorescence responses of the probe (1.0 μM) towards 1.0 μM of H_2S, GSH, and Cys, respectively. The black bars represent the addition of analyte without any pretreatment. The red bars and blue bars represent the pretreatment with DMSO at 60°C for 30 min and 60 min, respectively. Clearly, the pretreatment of GSH and Cys with DMSO greatly decrease their interference on the detection of hydrogen sulfide.

References

1. R. Kaushik, R. Sakla, A. Ghosh, G.T. Selvan, P. M. Selvakumar and D. A. Jose, *ACS Sens.* 2018**, 3**, 1142-1148.

2. S. Wang, H.C. Ding, Y.S. Wang, C.B. Fan, Y.Y. Tu, G. Liu and S.Z. Pu, *RSC Adv*., 2018**, 8,** 33121–33128.

3. X. F. Shanga, X. J. Yueb, Y.M. Chena, C. S. Lia, H. L. Chenc and T.Y. Wang, *Inorg. Chem. Commun.*, 2019, **99**, 1–10.

4. H. Fang, P. C. Huang and F. Y. Wu, *Spectrochim. Acta, Part A*, 2018, **204**, 568–57.

5. J. Y. Kim, S. Sarkar, K. N. Bobba, P. T. Huynh, A. Bhise and J. Yoo, *Org. Biomol. Chem*. 2019, DOI: 10.1039/c9ob00948e.

6. X. Li, Y. Gong, K. Wu, S.H. Liang, J. Cao, B. Yang, Y. Hu and Y. Han, *RSC Adv*. 2014, 4, 36106–36109.

7. M.G. Choi, S. Cha, H. Lee, H.L. Jeon and S. K. Chang, *Chem. Commun*. 2009, **47**, 7390–7392.

8. X. Gu, C. Liu, Y.C. Zhu and Y. Z. Zhu, *Tetrahedron Lett*. 2011, **52**, 5000–5003.

9. F. Hou, L. Huang, P. Xi, J. Cheng, X. Zhao, G. Xie, Y. Shi, F. Cheng, X. Yao, D. Bai, Z. Zeng, Inorg. Chem, 2012, **51**, 2454–2460.

10. S. Mirra, S. Milione, M. Strianese and C Pellecchia, *Eur. J. Inorg. Chem*. 2015, 2272–2276.
